# Twenty-year persistence of WT1-specific cytotoxic T lymphocytes in peritumoral brain tissue by peptide vaccine therapy: Transcriptomic evidence for resident memory T-cell induction

**DOI:** 10.1093/noajnl/vdag176

**Published:** 2026-07-09

**Authors:** Jun Nakata, Natsuki Nakamura, Daisuke Motooka, Chisato Yokota, Yoshiaki Hayashi, Ryuichi Hirayama, Fumihiro Fujiki, Soyoko Morimoto, Toru Umehara, Hiroko Nakajima, Yusuke Oji, Akihiro Tsuboi, Yoshihiro Oka, Atsushi Kumanogoh, Noriyuki Kijima, Haruhiko Kishima, Haruo Sugiyama

**Affiliations:** Department of Clinical Laboratory and Biomedical Sciences, Graduate School of Medicine, The University of Osaka, Osaka, Japan; Department of Neurosurgery, Graduate School of Medicine, The University of Osaka, Osaka, Japan; Genome Information Research Center, Research Institute for Microbial Diseases, Graduate School of Medicine, The University of Osaka, Osaka, Japan; Department of Neurosurgery, Graduate School of Medicine, The University of Osaka, Osaka, Japan; Genome Information Research Center, Research Institute for Microbial Diseases, Graduate School of Medicine, The University of Osaka, Osaka, Japan; Department of Neurosurgery, Graduate School of Medicine, The University of Osaka, Osaka, Japan; Department of Cancer Immunotherapy, Graduate School of Medicine, The University of Osaka, Osaka, Japan; Department of Cancer Stem Cell Biology, Graduate School of Medicine, The University of Osaka, Osaka, Japan; Department of Neurosurgery, Graduate School of Medicine, The University of Osaka, Osaka, Japan; Department of Cancer Immunology, Graduate School of Medicine, The University of Osaka, Osaka, Japan; Department of Clinical Laboratory and Biomedical Sciences, Graduate School of Medicine, The University of Osaka, Osaka, Japan; Department of Cancer Immunotherapy, Graduate School of Medicine, The University of Osaka, Osaka, Japan; Department of Cancer Stem Cell Biology, Graduate School of Medicine, The University of Osaka, Osaka, Japan; Department of Respiratory Medicine, Allergy and Rheumatic Disease, Graduate School of Medicine, The University of Osaka, Osaka, Japan; Department of Neurosurgery, Graduate School of Medicine, The University of Osaka, Osaka, Japan; Department of Neurosurgery, Graduate School of Medicine, The University of Osaka, Osaka, Japan; Department of Cancer Immunology, Graduate School of Medicine, The University of Osaka, Osaka, Japan

**Keywords:** glioma, oligoclonal expansion, resident memory T cells, tumor-infiltrating lymphocytes, WT1 peptide vaccine

## Abstract

**Background:**

Sustained immune surveillance at tumor sites is considered essential for effective cancer immunotherapy; however, direct evidence of immune surveillance in humans is limited due to difficulty obtaining tumor and peritumoral tissues through invasive procedures.

**Methods:**

Peritumoral brain tissue was obtained during reoperation from a patient with *diffuse astrocytoma* who had received continuous Wilms’ tumor gene 1 (WT1) peptide vaccination for 20 years. WT1 tetramer-positive CD8+ T cells were analyzed by flow cytometry, followed by T-cell receptor repertoire analysis and single-cell RNA sequencing.

**Results:**

A high level of T-cell infiltration was observed in peritumoral brain tissue, despite the immune-privileged nature of the central nervous system. Notably, 38.4% of CD8^+^  T cells were WT1-tetramer positive, exhibiting an oligoclonal pattern similar to that observed in peripheral blood. Single-cell RNA sequencing demonstrated that WT1-specific cytotoxic T lymphocytes (CTLs) were predominantly composed of resident memory T cells (Trm) and terminally differentiated effector memory T cells (TEMRA), along with a small fraction of cycling T cells. Interestingly, Trm-like subsets were also detected among WT1-specific CTLs in peripheral blood.

**Conclusion:**

These findings provide in situ evidence of long-term persistence of WT1-specific CTLs in the human brain by continuous WT1 peptide vaccination. Such local immune surveillance may have contributed to the prevention of relapse in this patient. Single-cell RNA sequencing suggests that long-lived Trm may contribute to the persistence of immune surveillance through self-renewal and generation of cytotoxic TEMRA. This study offers insights into long-term anti-tumor immunity and may inform future optimization of cancer vaccine therapy.

Key Points

- High infiltration of WT1-specific CTLs was observed in peritumoral brain tissues of a glioma patient after 20 years of continuous WT1 peptide vaccination.
- Expanded clones were mainly Trm and TEMRA, with a minor fraction of proliferating cells.

Importance of the Study

This study provides rare in situ evidence of long-term persistence of tumor antigen-specific T cells within the human brain following continuous WT1 peptide vaccination. We obtained peritumoral tissue during reoperation for a newly developed lesion, allowing direct analysis of the local immune environment. WT1-specific cytotoxic T lymphocytes (CTLs) with an oligoclonal pattern were persistently present in peritumoral tissue even after 20 years of continuous vaccination. Single-cell RNA sequencing further revealed that WT1-specific CTLs were predominantly composed of resident memory T cells (Trm) and terminally differentiated effector memory T cells (TEMRA), along with a small fraction of cycling T cells. These findings suggest that long-lived Trm may undergo self-renewal while generating highly cytotoxic TEMRA, thereby sustaining immune surveillance in the peritumoral brain. Our results contribute to a better understanding of long-term anti-tumor immunity in glioma and may inform the development of cancer vaccine strategies.

Glioma remains a fatal disease due to high recurrence rates, even after multimodal treatment with surgery, radiotherapy, and chemotherapy. Cancer immunotherapy, which is now an established therapeutic option in several malignancies, is therefore attracting attention as a potential strategy for glioma. Among these approaches, cancer vaccine therapy,^1-3^ immune checkpoint inhibitors, and chimeric antigen receptor T-cell therapy^4^ are currently in advanced stages of clinical development. Of note, we have experienced long-term survivors with glioma receiving continuous Wilms’ tumor gene 1 (WT1) peptide vaccinations. We previously reported that WT1 peptide vaccination promoted high infiltration of WT1-specific CTLs and converted glioma into a “hot tumor” resulting in improved survival when combined with checkpoint inhibitors in a murine model.^5^ However, direct assessment of such immune phenomena in humans has been challenging due to the invasiveness of sampling brain tissue. Here, we describe a unique long-term survivor treated with continuous WT1 peptide vaccinations, in whom peritumoral brain tissue was available for immunological analysis, thereby providing rare evidence of sustained immune surveillance in the human brain.

## Case Presentation

**Figure 1.**
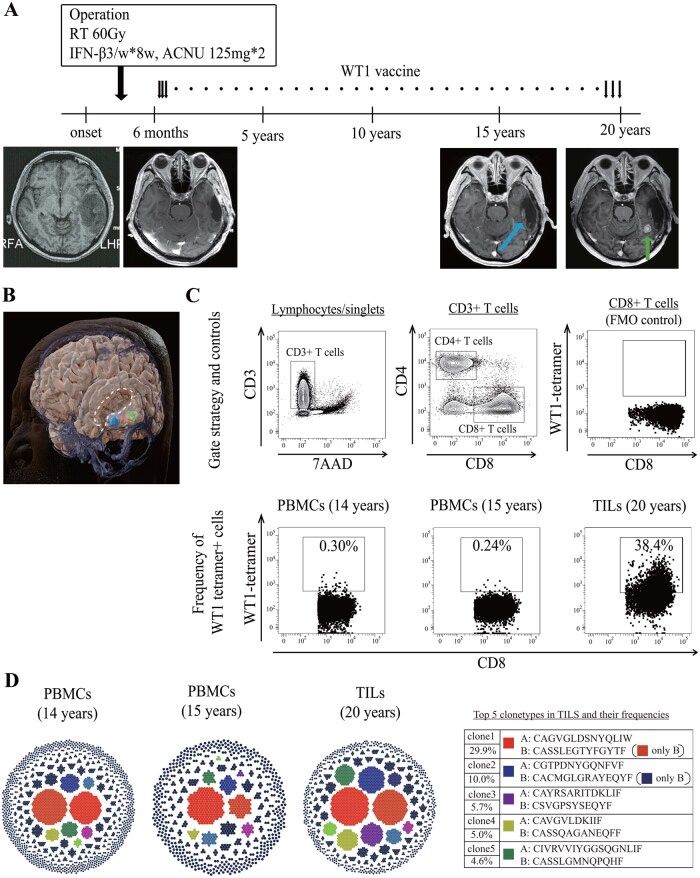
Clonal distribution of WT1-specific CTLs in peritumoral brain tissue. (A) Clinical timeline of the patient. Arrows indicate the contrast-enhanced lesions that appeared from 15 and 20 years after onset, respectively. (B) Three-dimensional reconstructed image used for surgical planning. Blue and green areas correspond to the contrast-enhanced lesions indicated by arrows of the same color in (A), and the intervening orange area was collected for immunological analysis. (C) Flow cytometry analysis of WT1 tetramer-positive CD8^+^T cells. The upper panels show the gating strategy and WT1 tetramer staining with FMO control. The lower panels show representative plots of WT1 tetramer-PE versus CD8-Alexa647 for 7AAD-CD3^+^CD8^+^ T cells from PBMCs collected 14 and 15 years after onset, and from tumor-infiltrating lymphocytes (TILs) obtained 20 years after onset. The frequency of WT1 tetramer-positive cells is indicated in each plot. (D) Clone distribution maps of CD3^+^CD8^+^WT1-tetramer^+^ T cells gated in (C) from PBMCs at 14 and 15 years and from TILs at 20 years. Each dot represents a single cell, and cells sharing identical TCR sequences form clusters. The accompanying table shows the amino acid sequences of the CDR3 regions of TCRα and TCRβ for the dominant clonotypes, which are defined based on TCRβ sequence, together with their relative frequencies (%) within the analyzed cells. Cells lacking detectable paired TCRα sequences but sharing identical TCRβ sequences were included within the corresponding clonotypes and are indicated as “only B” in the table.

A 55-year-old man presented with writing difficulty in February 2005 and was diagnosed with an *anaplastic astrocytoma* of the left lateral ventricle (Figure 1A). Subsequent immunohistochemical analysis revealed an IDH1 mutation, and the tumor is currently classified as *diffuse astrocytoma*, *IDH-mutant*, *WHO Grade 2* under the WHO 2021 classification. He underwent awake craniotomy, followed by radiotherapy (expanded local field, total 60 Gy) and adjuvant therapy with weekly Interferon-β for 8 weeks and 2 courses of nimustine. The patient subsequently enrolled in the “Phase I/II clinical trial of WT1 peptide-based vaccine for the patients with malignant tumors” (UMIN 000002001), and received the WT1-235 peptide vaccine weekly for 12 weeks according to the study protocol.^1^ MRI evaluation after the 12-week protocol showed stable disease, with only the postoperative cavity and no contrast enhancement, and long-term continuation of WT1 vaccination was initiated in line with the study protocol. As the initial Phase I/II trial approached its long-term limit, the IRB recommended transitioning to a new study designed to evaluate prolonged safety, and in 2014 he was switched to enroll in “WT1 peptide-based cancer vaccine for long-term maintenance of anti-tumor immunity” (UMIN000015997), under which WT1 vaccination was further continued every 3 months. In total, he received approximately 150 vaccinations over a 20-year period.

During the 20-year follow-up, he remained free from neurological sequelae except for post-operative anterograde amnesia, with an estimated Karnofsky Performance Status of 70-80. In 2020, however, a small contrast-enhanced lesion appeared on the dorsal side of the resection cavity (Figure 1 A, 15-year MRI). As the lesion remained stable, he continued WT1 vaccination with MRI monitoring every 3 months. In 2025, a new enhancing nodule emerged adjacent to the previous one (Figure 1A, 20-year MRI). Because tumor recurrence could not be excluded, repeat surgery was performed in February 2025. For the second surgery, 3D reconstruction was performed to visualize the operative field and highlight the lesion with color (Figure 1B). Both enhancing lesions (blue and green in Figure 1B) were resected, and intervening small tissue fragments (orange in Figure 1B), normally discarded during the surgical approach, were collected separately for immunological analysis. Histopathology revealed that the green lesion was a benign hemangioma, and no residual tumor was identified in the blue lesion.

### Oligoclonal Expansion of WT1-Specific CTLs Within Peritumoral Brain Tissue

Although normal brain parenchyma usually contains very few immune cells due to the blood-brain barrier, a large number of CD45^+^ cells were recovered from the small peritumoral fragments (orange in Figure 1B). Flow cytometry revealed that the majority of these cells were T lymphocytes. Notably, CD8^+^ T cells showed a clear positive shift in PE fluorescence intensity for the WT1 tetramer, and as many as 38.4% of CD8^+^ T cells were within the WT1 tetramer-positive gate defined by the fluorescence minus one (FMO) control (Figure 1C). By contrast, the frequencies of WT1 tetramer-positive CD8^+^ T cells in frozen PBMCs from 2019 and 2020 were 0.30% and 0.24%, respectively (Figure 1C). These levels are higher than those observed in healthy individuals and are consistent with high frequencies in patients with a favorable clinical course treated with WT1 peptide vaccination.^1^ Taken together, these results indicate that an exceptionally high accumulation of WT1 tetramer-positive CD8^+^ T cells was observed in peri-tumoral tissue of the patient.

To further confirm the antigen specificity of the WT1 tetramer-positive CD8^+^ T cells, we performed TCR repertoire and single-cell RNA sequencing analyses on WT1 tetramer-positive CD8^+^ T cells from both peritumoral tissue and frozen PBMCs. TCR repertoire analysis demonstrated a clear oligoclonal pattern among WT1 tetramer-positive CD8^+^ T cells in peritumoral tissue (Figure 1D). The dominant clonotype, defined by CDR3β sequence CASSLEGTYFGYTF, accounted for 29.9% of the population, followed by CACMGLGRAYEQYF, CSVGPSYSEQYF, CASSQAGANEQFF, and CASSLGMNQPQHF, together comprising 55.1% of the population. In addition, some cells lacked identifiable TCRα sequences but shared identical TCRβ sequences with these dominant clonotypes and therefore were included as “only B” in the accompanying table in Figure 1D, suggesting that they likely represented the same clonotypes. A recent report demonstrated that such marked oligoclonality is absent in healthy individuals but is consistent with the characteristic feature of long-term responders treated with WT1 peptide vaccination.^6^ Importantly, all of these top 5 clones were also detected in frozen PBMCs obtained in both 2019 and 2020, indicating that these WT1-specific CTL clones had been stably maintained for more than 5 years during continuous WT1 peptide vaccination.

### Trm Subsets With a Few Dividing T Cells Within Expanded WT1-Specific CTL Clones in Peritumoral Tissue

**Figure 2.**
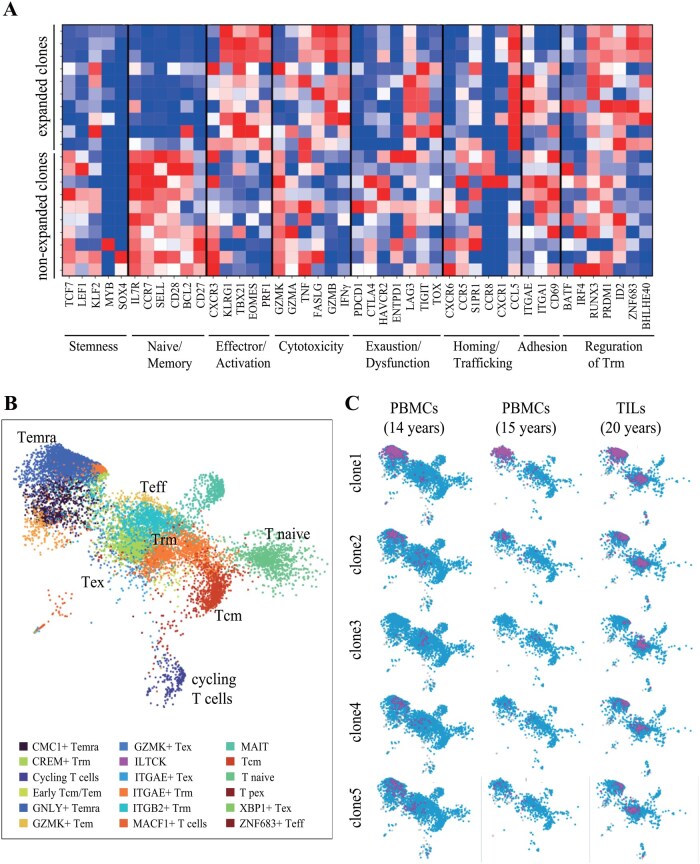
Trm subsets with a few dividing T cells within expanded WT1-specific CTL clones in peritumoral tissue. (A) Heatmap comparison of gene expression between expanded and non-expanded clones. Expanded clones correspond to the top 10 most abundant clones, highlighted in color in Figure 1E. Non-expanded clones were randomly grouped into 10 sets, each comprising cells from clones with ≤10 cells in Figure 1E, to allow balanced visualization. (B) UMAP projection of CD3^+^CD8^+^WT1-tetramer^+^ T cells from PBMCs (14 and 15 years after onset) and TILs (20 years after onset). Cell-type annotation was performed by scANVI based on reference integration. (C) Distribution of the top 5 expanded clones in Figure 1D, visualized by overlaying clone-specific cells (pink) on the UMAP background of all WT1-specific CTLs (blue) for each sample and time point.

To clarify how these WT1-specific CTL clones had been maintained for more than 5 years, we first compared the transcriptional profiles of expanded and non-expanded WT1-specific CTL clones in peritumoral tissue using RNA sequencing data. Non-expanded clones showed higher expression of stemness-related genes (TCF7, LEF1, KLF2) and naïve/memory-associated genes (IL7R, CCR7, SELL, CD28, BCL2, CD27) (Figure 2A). By contrast, expanded clones exhibited higher expression of effector/activation genes (KLRG1, TBX21, EOMES, PRF1) and cytotoxicity-related genes (FASLG, GZMB, IFNG). These findings indicate that expanded clones possessed stronger effector functions and the potential to eliminate WT1-expressing target cells, thereby contributing to immunosurveillance. Although these clones exhibited strong cytolytic activity, they did not show upregulation of exhaustion-related genes such as PDCD1 or CTLA4. Instead, they expressed high levels of ZNF683 and BHLHE40, which are implicated in the regulation and maintenance of resident memory. Furthermore, CCL5 associated with trafficking was consistently higher in expanded clones than in non-expanded clones.

To further investigate why expanded WT1-specific CTLs exhibited both strong effector function and resident memory characteristics, we performed UMAP clustering analysis of WT1-specific CTLs FACS-sorted from peritumoral tissue and frozen PBMCs in 2019 and 2020, as gated in Figure 1C. We performed single-cell gene expression and TCR repertoire analysis on approximately 3,000 cells per sample. We integrated our data with a public reference huARdb database (∼440,000 human single-cell immune profiling cells)^7^ and trained an scVI model to create a joint latent space. We then used scANVI to transfer cell-type labels to our data. Using this annotation, we generated an UMAP and performed clustering on our dataset (Figure 2B). Next, the distribution of the top 5 expanded clones identified in Figure 1D was visualized on the UMAP of WT1-specific CTLs for each sample (Figure 2C). In the peritumoral tissue, all top 5 clones were predominantly localized within Trm and TEMRA clusters, with the relative population differing among clones. Importantly, each clone also contained a subset of cycling T cells, indicating that proliferative activity was present within these long-term persisting clones. In PBMCs, all top 5 clones were predominantly localized within TEMRA clusters. However, a few cells from each clone were also localized within Trm clusters.

## Discussion

To our knowledge, this is the first report demonstrating tumor-associated antigen (TAA)-specific CTLs persisting in peritumoral tissue for over 2 decades. Despite the brain being an immune-privileged site, we observed high infiltration of T cells, including oligoclonally expanded WT1-specific CTLs. Since gliomas typically relapses in the vicinity of the primary site,^8^^,^^9^ such durable CTL infiltration likely contributed to preventing recurrence. These findings clearly indicate that continuous WT1 peptide vaccination can maintain immune surveillance within the peritumoral compartment. Direct evaluation of tumor sites has not been reported, but several studies have shown that sustained TAA-specific CTLs can be detected in PB of long-term survivors receiving cancer peptide vaccinations.^10-12^ The frequencies of TAA-specific CTLs in PB are generally modest, typically <1.0% in these studies and in our data, because PB captures them only in transit within the broad pool of circulating T cells, whereas local tissues reflect their cumulative accumulation over time. Indeed, both murine tumor model and human graft-versus-host disease (GVHD) have demonstrated that CTL clones <1.0% in PB can dominate at around 20% within tumor sites and GVHD organs, respectively.^13^^,^^14^ In line with these observations, the detection of WT1-specific CTLs at 0.30% and 0.24% in PB in our patient corresponded to a striking 38.4% accumulation in peritumoral brain tissue. Our results strongly support previous findings that even modest frequencies of TAA-specific CTLs in PB can be important predictors of clinical efficacy.

Transcriptomic analysis of TAA-specific CTLs in long-survivors has been difficult because of their low numbers and biased subset composition. In our study, reference integration and scANVI enabled robust annotation, clarifying that Trm and TEMRA were the dominant subsets in peritumoral brain tissue. Trm are long-lived, locally resident memory T cells, originally characterized in mucosal immunity,^15^ and their tumor infiltration has been associated with better survival across multiple tumor types.^16^^,^^17^ Furthermore, tumor-infiltrating Trm have recently been linked to favorable response of immunotherapy including checkpoint inhibitors and cancer vaccine therapy. Several mouse studies demonstrated that induction of Trm is crucial for the efficacy of cancer vaccines, and novel vaccination routes have been proposed to enhance their generation.^18^ Taken together, prominent infiltration of Trm subsets among WT1-specific CTL clones in our patient likely contributed to his long-term survival without recurrence. Since expanded WT1-specific CTL clones included cycling T cells, suggesting ongoing proliferation in situ, Trm may differentiate into highly cytotoxic TEMRA while simultaneously replenishing the Trm pool through self-renewal. In addition, Trm-like subsets were also detected in PB in our analysis. Recent reports suggest that a small fraction of Trm can re-differentiate and recirculate as so-called ex-Trm cells.^19^^,^^20^ Although their functional role remains to be clarified, such TAA-specific ex-Trm subsets in PB raise the possibility that they could serve as peripheral biomarkers of effective vaccine response.

Elucidating the biology of Trm, particularly their induction and long-term maintenance, will be crucial for advancing cancer vaccine therapy. Broader transcriptomic analyses are needed; for example, in this case we noted markedly higher CCL5 expression in expanded clones, together with well-established Trm-associated genes such as ITGAE, ZNF683, and BHLHE40. CCL5 is well known in cancer immunity for its tumor cell-derived immunosuppressive function through Treg recruitment,^21^ but it can also act in the opposite direction by promoting immune activation depending on the context.^22^ Recent studies in murine autoimmune models have shown that long-resident Trm can sustain local inflammation and recruit circulating effector T cells through CCL5 production.^23^^,^^24^ Although this mechanism has not yet been demonstrated in tumor immunity, it is plausible that the peritumoral Trm persisting for over 2 decades in our patient may have played a similar role. Further transcriptomic studies of TAA-specific CTL clones in both PB and tumor sites of long-term survivors will be essential to clarify the mechanisms underlying Trm induction, regulation, and maintenance in cancer vaccine therapy.

## Data Availability

The data that support the findings of this study are available from the corresponding author upon reasonable request.

## References

[1] Izumoto S , Tsuboi A, Oka Y, et al Phase II clinical trial of Wilms tumor 1 peptide vaccination for patients with recurrent glioblastoma multiforme. J Neurosurg. 2008;108:963-971. 10.3171/JNS/2008/108/5/0963 18447714

[2] Tsuboi A , Hashimoto N, Fujiki F, et al A phase I clinical study of a cocktail vaccine of Wilms’ tumor 1 (WT1) HLA class I and II peptides for recurrent malignant glioma. Cancer Immunol Immunother. 2019;68:331-340. 10.1007/s00262-018-2274-1 30430205 PMC6394509

[3] Hashimoto N , Tsuboi A, Kagawa N, et al Wilms tumor 1 peptide vaccination combined with temozolomide against newly diagnosed glioblastoma: safety and impact on immunological response. Cancer Immunol Immunother. 2015;64:707-716. 10.1007/s00262-015-1674-8 25772149 PMC11028974

[4] Brown CE , Hibbard JC, Alizadeh D, et al Locoregional delivery of IL-13Rα2-targeting CAR-T cells in recurrent high-grade glioma: a phase 1 trial. Nat Med. 2024;30:1001-1012. 10.1038/s41591-024-02875-1 38454126 PMC11031404

[5] Yokota C , Nakata J, Takano K, et al Distinct difference in tumor-infiltrating immune cells between Wilms’ tumor gene 1 peptide vaccine and anti-programmed cell death-1 antibody therapies. Neurooncol Adv. 2021;3:vdab091. 10.1093/noajnl/vdab091 34355173 PMC8331049

[6] Morimoto S , Tanaka Y, Nakata J, et al Spontaneous high clonal expansion of Wilms’ tumor gene 1-specific cytotoxic T-lymphocytes in patients with Wilms’ tumor gene 1-expressing solid tumor. Cancer Immunol Immunother. 2024;74:15. 10.1007/s00262-024-03862-8 39509060 PMC11543974

[7] Wu L , Xue Z, Jin S, et al huARdb: human antigen receptor database for interactive clonotype-transcriptome analysis at the single-cell level. Nucleic Acids Res. 2022;50:D1244-D1254. 10.1093/nar/gkab857 34606616 PMC8728177

[8] Tu Z , Xiong H, Qiu Y, Li G, Wang L, Peng S. Limited recurrence distance of glioblastoma under modern radiotherapy era. BMC Cancer. 2021;21:720. 10.1186/s12885-021-08467-3 34154559 PMC8218451

[9] Brandes AA , Tosoni A, Franceschi E, et al Recurrence pattern after temozolomide concomitant with and adjuvant to radiotherapy in newly diagnosed patients with glioblastoma: correlation with MGMT promoter methylation status. J Clin Oncol. 2009;27:1275-1279. 10.1200/JCO.2008.19.4969 19188675

[10] Kjeldsen JW , Iversen TZ, Engell-Noerregaard L, Mellemgaard A, Andersen MH, Svane IM. Durable clinical responses and long-term follow-up of stage III-IV non-small-cell lung cancer (NSCLC) patients treated with IDO peptide vaccine in a phase I study: a brief research report. Front Immunol. 2018;9:2145. 10.3389/fimmu.2018.02145 30283461 PMC6157336

[11] Ellingsen EB , Aamdal E, Guren T, et al Durable and dynamic hTERT immune responses following vaccination with the long-peptide cancer vaccine UV1: long-term follow-up of three phase I clinical trials. J Immunother Cancer. 2022;10:e004345. 10.1136/jitc-2021-004345 PMC9134181 35613827

[12] Mizukoshi E , Nakagawa H, Tamai T, et al Peptide vaccine-treated, long-term surviving cancer patients harbor self-renewing tumor-specific CD8. Nat Commun. 2022;13:3123. 10.1038/s41467-022-30861-z 35660746 PMC9166698

[13] Nakata J , Nakajima H, Hayashibara H, et al Extremely strong infiltration of WT1-specific CTLs into mouse tumor by the combination vaccine with WT1-specific CTL and helper peptides. Oncotarget. 2018;9:36029-36038. 10.18632/oncotarget.26338 30542516 PMC6267595

[14] Koyama D , Murata M, Hanajiri R, et al Quantitative assessment of T cell clonotypes in human acute graft-versus-host disease tissues. Biol Blood Marrow Transplant. 2019;25:417-423. 10.1016/j.bbmt.2018.10.012 30359734

[15] Kaufman DR , Liu J, Carville A, et al Trafficking of antigen-specific CD8+ T lymphocytes to mucosal surfaces following intramuscular vaccination. J Immunol. 2008;181:4188-4198. 10.4049/jimmunol.181.6.4188 18768876 PMC2580672

[16] Webb JR , Milne K, Watson P, Deleeuw RJ, Nelson BH. Tumor-infiltrating lymphocytes expressing the tissue resident memory marker CD103 are associated with increased survival in high-grade serous ovarian cancer. Clin Cancer Res. 2014;20:434-444. 10.1158/1078-0432.CCR-13-1877 24190978

[17] Djenidi F , Adam J, Goubar A, et al CD8+CD103+ tumor-infiltrating lymphocytes are tumor-specific tissue-resident memory T cells and a prognostic factor for survival in lung cancer patients. J Immunol. 2015;194:3475-3486. 10.4049/jimmunol.1402711 25725111

[18] Nizard M , Roussel H, Diniz MO, et al Induction of resident memory T cells enhances the efficacy of cancer vaccine. Nat Commun. 2017;8:15221. 10.1038/ncomms15221 28537262 PMC5458068

[19] Fonseca R , Beura LK, Quarnstrom CF, et al Developmental plasticity allows outside-in immune responses by resident memory T cells. Nat Immunol. 2020;21:412-421. 10.1038/s41590-020-0607-7 32066954 PMC7096285

[20] Rodger B , Stagg AJ, Lindsay JO. The role of circulating T cells with a tissue resident phenotype (ex-T). Front Immunol. 2024;15:1415914. 38817613 10.3389/fimmu.2024.1415914 PMC11137204

[21] Chang LY , Lin YC, Mahalingam J, et al Tumor-derived chemokine CCL5 enhances TGF-β-mediated killing of CD8(+) T cells in colon cancer by T-regulatory cells. Cancer Res. 2012;72:1092-1102. 10.1158/0008-5472.CAN-11-2493 22282655

[22] Schall TJ , Bacon K, Toy KJ, Goeddel DV. Selective attraction of monocytes and T lymphocytes of the memory phenotype by cytokine RANTES. Nature. 1990;347:669-671. 10.1038/347669a0 1699135

[23] Gao A , Zhao W, Wu R, et al Tissue-resident memory T cells: the key frontier in local synovitis memory of rheumatoid arthritis. J Autoimmun. 2022;133:102950. 10.1016/j.jaut.2022.102950 36356551

[24] Steinbach K , Vincenti I, Egervari K, et al Brain-resident memory T cells generated early in life predispose to autoimmune disease in mice. Sci Transl Med. 2019;11:eaav5519. 10.1126/scitranslmed.aav5519 31243152

